# Salivary uric acid remote serial self‐testing for prediction of adverse pregnancy outcomes of uteroplacental dysfunction

**DOI:** 10.1111/aogs.70257

**Published:** 2026-05-14

**Authors:** Basia Chmielewska, Chao Wang, Nick Macklon, Amar Bhide, Basky Thilaganathan

**Affiliations:** ^1^ Fetal Medicine Unit St George's University Hospitals NHS Foundation Trust London UK; ^2^ Vascular Biology Research Centre, Molecular and Clinical Sciences Research Institute City St George's University of London London UK; ^3^ Kingston University London UK; ^4^ London Women's Clinic London UK; ^5^ Zealand University Hospital Roskilde Denmark

**Keywords:** fetal growth restriction, hypertensive disorders of pregnancy, remote monitoring, uric acid

## Abstract

**Introduction:**

Hypertensive disorders of pregnancy (HDP) and fetal growth restriction (FGR) are major manifestations of uteroplacental dysfunction and remain leading causes of maternal and perinatal morbidity. Current strategies for risk assessment rely on hospital‐based screening or diagnostic triage and do not provide continuous, personalized monitoring throughout pregnancy. Uric acid is closely linked to placental dysfunction, and salivary uric acid (sUA) reflects circulating concentrations, enabling noninvasive remote measurement. This study evaluated whether serial, smartphone‐enabled self‐testing of sUA could predict adverse pregnancy outcomes related to uteroplacental dysfunction.

**Material and Methods:**

In this prospective observational cohort study, pregnant women were recruited at 20–22 weeks' gestation at a tertiary hospital. Participants performed weekly salivary self‐testing using colorimetric uric acid strips until delivery, with results captured via a smartphone application. Images were analyzed using RGB color metrics. Primary outcomes were HDP and/or FGR, defined using ISSHP and FIGO criteria, respectively. Logistic regression models incorporated 4 weeks of lagged RGB‐derived sUA metrics together with maternal characteristics. Model performance was assessed using area under the receiver operating characteristic curve (AUC) and calibration metrics, stratified by gestational age at outcome.

**Results:**

Of 495 recruited participants, 318 women with complete longitudinal data were included in the final analysis; 36 (11.3%) developed HDP and/or FGR. A multivariable model combining maternal risk factors with 4 weeks of serial sUA measurements demonstrated good predictive performance. Discrimination was highest for preterm outcomes, with an AUC of 0.814 for prediction within 1 week at <37 weeks' gestation and prediction remained acceptable at term (AUC 0.732).

**Conclusions:**

Remote serial self‐testing of salivary uric acid is feasible and shows good predictive ability for adverse pregnancy outcomes related to uteroplacental dysfunction. This noninvasive approach has potential to complement existing screening and surveillance strategies by enabling continuous, personalized risk assessment throughout pregnancy. Prospective validation in larger and more diverse populations is warranted.

## Graphical abstract

Remote, weekly self‐testing of salivary uric acid enables longitudinal monitoring throughout pregnancy. RGB‐derived measurements are combined with maternal characteristics in a model using 4 weeks of prior data. This approach provides individualized prediction of hypertensive disorders of pregnancy and fetal growth restriction within 1 week. External validation is required.

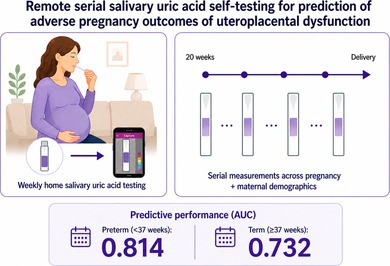

**Abbreviations**
AUC: area under the curve
FGR: fetal growth restriction
HDP: hypertensive disorders of pregnancy
RGB: Red–Green–Blue
ROC: receiver operating characteristic
sUA: salivary uric acid

**Key message**

Serial salivary uric acid measurements obtained through smartphone‐enabled self‐testing can be used in prediction of hypertensive disorders of pregnancy and/or fetal growth restriction. The findings of this study support the concept of pregnancy surveillance models that shift from episodic assessment to longitudinal, patient‐generated data.

## 1 INTRODUCTION

Hypertensive disorders of pregnancy (HDP) and fetal growth restriction (FGR) represent key clinical manifestations of uteroplacental dysfunction and remain major drivers of maternal and perinatal morbidity worldwide.^1^, ^2^ Current approaches to risk assessment for HDP (including preeclampsia) and FGR are confined to two distinct contexts: screening versus diagnostic triage. Elective screening strategies, including the first‐trimester Fetal Medicine Foundation (FMF) algorithm and checklist‐based approaches such as those recommended by National Institute for Health and care Excellence (NICE), to stratify women into binary risk categories principally using demographics and medical history, but also include biomarkers and ultrasound parameters.^3^, ^4^, ^5^ Diagnostic triage is used in symptomatic women or those with suspected HDP/FGR, typically using angiogenic biomarkers such as sFlt‐1/PlGF, to assist with short‐term risk stratification and clinical decision‐making.^4^ Both strategies rely on hospital‐based clinical assessment, significant laboratory infrastructure and focus mainly on preterm pathology. A tool to provide personalized, noninvasive, remote and continuous risk assessment for HDP and FGR throughout pregnancy, is currently unavailable.

Uric acid, the end product of purine metabolism, has long been associated with HDP and FGR of uteroplacental origin through its link to oxidative stress, a central feature of endothelial dysfunction, placental hypoxia and the maternal systemic inflammatory response that characterizes placental insufficiency.^6^, ^7^, ^8^, ^9^ Data suggest that uric acid may act, not only as a marker, but also as a mediator of disease, thereby linking maternal vascular pathology with impaired fetal growth.^10^, ^11^, ^12^ Maternal serum uric acid concentrations are reflected in saliva, enabling measurement through noninvasive sampling methods.^9^, ^13^, ^14^, ^15^ Salivary uric acid (sUA) has therefore been proposed as a potential biomarker in identifying pregnancies complicated by uteroplacental dysfunction.^15^ A recent prospective cohort study demonstrated that remote sUA testing was feasible and that elevated sUA levels preceded the development of HDP and FGR, indicating proof of concept for a longitudinal predictive approach.^16^ This study extends on the latter finding by testing whether a longitudinal model based on several weeks of lagged sUA measurements, combined together with maternal risk factors, can predict upcoming adverse pregnancy outcomes related to uteroplacental dysfunction.

## 2 MATERIAL AND METHODS

This prospective study uses data from a previously collected cohort to evaluate whether serial, smartphone‐based sUA self‐testing could be used in a time‐updated prediction model for HDP and FGR^16^ The cohort was collected at Zealand University Hospital, Denmark, between August 2017 and July 2018. Participants were considered eligible for recruitment if they were between 20^+0^ and 21^+6^ weeks' gestation, aged 18 years or older, able to communicate in Danish or English, and had access to a smartphone. Pregnancies in which fetal structural malformations were identified on ultrasound were excluded. Pregnant women attending their routine mid‐trimester anomaly scan were approached for participation. Each received written and verbal information and provided informed consent before enrollment. The study was approved by the Regional Committee on Health Research Ethics (SJ‐583), the Danish Data Protection Agency (REG‐140‐2018), and the Danish Medicines Agency (2016100924).

### 2.1 Sample collection

Each participant received a box containing self‐sampling swabs with colorimetric uric acid test strips and received training on how to perform saliva self‐sampling. They were instructed to perform a test once a week until delivery, first thing in the morning, prior to exercise, smoking, or consumption of food or drink. The test strips utilized a reaction in which uric acid reduces Cu^2+^ to Cu^+^, generating a sodium bicinchoninate–Cu^+^ purple chelate, thereby producing a visible purple color change. Participants used a smartphone application to photograph each test strip. Photos were taken against the standardized background of the provided box, which displayed the participant's study ID and a fixed Red–Green–Blue (RGB) color reference to standardize image analysis. Digital images were processed using MATLAB (MathWorks Inc., Massachusetts, USA). The shade and intensity of the purple chelate were converted into quantitative color metrics using the RGB color model in which each pixel is represented by three intensity values (red, green, and blue) ranging from 0 to 255. For each sample, both the mean RGB value (RGB_mean) and the minimum RGB value (RGB_min) were calculated across all pixels and used in statistical analysis. The smartphone application reminded participants to complete testing and allowed participants to submit images of the test.

### 2.2 Outcome measures

The primary outcomes were HDP and FGR. HDP was defined using International Society for the Study of Hypertension in Pregnancy (ISSHP) criteria, including gestational hypertension (blood pressure ≥ 140/90 mmHg after 20 weeks without proteinuria) and preeclampsia (hypertension after 20 weeks with proteinuria, maternal organ dysfunction, or uteroplacental dysfunction). FGR was defined according to FIGO, as estimated fetal weight or abdominal circumference below the 10th percentile for gestational age, often with abnormal fetal and/or uterine Doppler findings indicative of placental insufficiency.^2^, ^17^ Baseline demographic and clinical characteristics were recorded at recruitment. Pregnancy and neonatal outcomes were obtained from hospital electronic medical records following delivery. Testing compliance was calculated as the number of tests completed divided by the number of qualifying study weeks, expressed as a percentage.

### 2.3 Statistical analysis

The sample size of 500 participants was estimated using an assumed 10% prevalence of relevant clinical outcomes (HDP/FGR), 80% expected compliance, and a requirement to capture 40 events. Data from this cohort have been published previously focusing on descriptive analyses^16^ The present analysis was designed to move beyond feasibility and descriptive reporting by testing whether repeated weekly RGB‐derived sUA measurements, combined with maternal characteristics, could predict imminent adverse outcomes. A prediction model was developed by training a logistic regression model incorporating colorimetric metrics such as RGB_mean and RGB_min prior to the defined outcome, as well as women's characteristics such as maternal BMI, maternal age, smoking status, prior PE, and gestational week.

To determine the optimal number of weeks' colorimetric value before the outcome event to be used in the prediction model, a series of logistic regression models were tested using a varying number of lagged RGB variables. Of note, the training sample size became smaller as more lagged RGB variables were used due to both the nature of dynamic modeling and missing data (listwise deletion used). Tjur's *R* ^2^ was used to assess the prediction performance and finalize the model specification, as pseudo‐*R* ^2^ measures the overall performance encapsulating both discrimination and calibration aspects of a model.^18^ The optimal number was found to be 4 weeks' lag of RGB variables, which was used in the final prediction model. Logistic regressions with the Firth correction were used as it demonstrates good performance particularly in small datasets.^19^ The modeling performance was evaluated using common approaches such as the area under the receiver operating characteristic (ROC) curve and area under the curve (AUC) and calibration plots for prediction before and after 37 weeks separately.

## 3 RESULTS

A total of 495 women were recruited to the study, with a mean compliance rate of 67% (range 0%–100%) for weekly testing from 22 weeks' gestation. Excluding those without a complete 4‐week sequence of measurements prior to birth or an adverse pregnancy outcome, 318 participants were included in the final logistic regression analysis. Among these, 36 participants (11.3%) developed a relevant clinical outcome (HDP or FGR). Baseline characteristics were broadly similar between affected and unaffected participants, although prior preeclampsia was more common among those with an adverse pregnancy outcome (13.9% vs. 1.4%) (Table 1).

**TABLE 1:** Maternal demographic characteristics in the study participants with normal and adverse pregnancy outcomes.

	Normal outcome	HDP and/or FGR	*p*‐value
	282 (88.7%)	36 (11.3%)	
Maternal age (years)	31.2 (4.8)	31.5 (4.7)	0.731
BMI	24.8 (4.8)	28.1 (6.2)	0.002
Smokers	12 (4.3%)	0 (0.0%)	0.207
Previous preeclampsia	4 (1.4%)	5 (13.9%)	<0.001
Gestation of HDP and/or FGR diagnosis (week)	–	37 + 6 (35 + 6 – 39 + 3)	–
<37^+0^ weeks	–	12 (33.3%)	–
≥37^+0^ weeks	–	24 (66.7%)	–

*Note*: Data are shown as mean (standard deviation) for parametric variables, number (%) for categorical variables and median (interquartile range) for nonparametric variables.

Abbreviations: BMI, body mass index; FGR, fetal growth restriction; HDP, hypertensive disorder of pregnancy.

**FIGURE 1:**
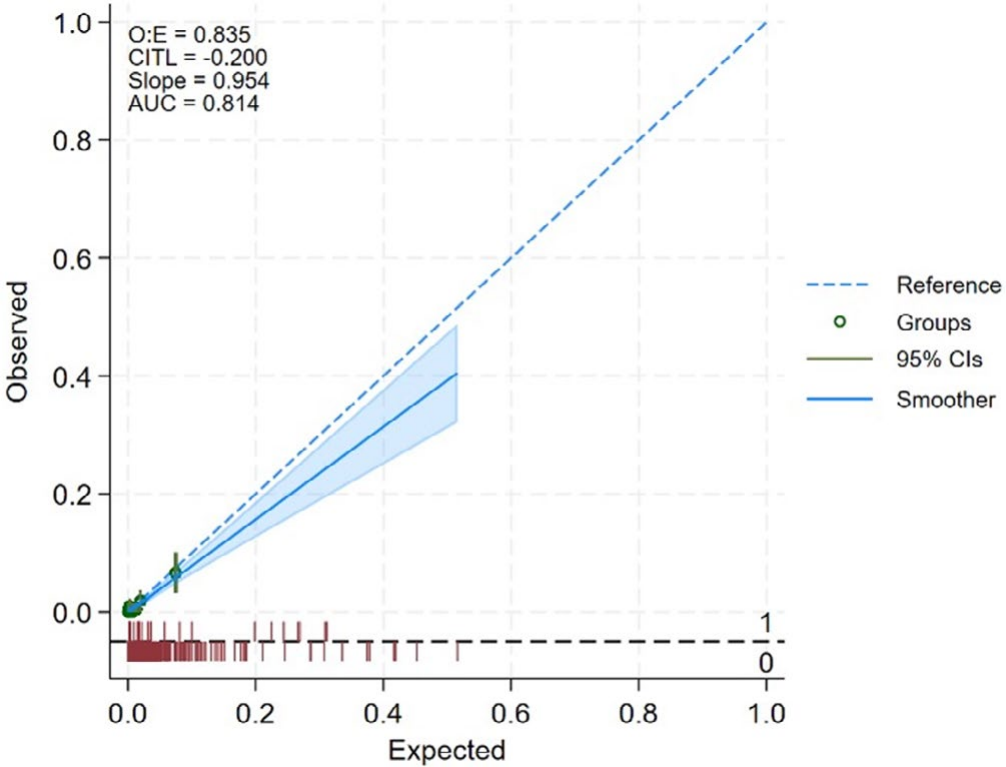
Prediction of preterm HDP/FGR within 1 week. Calibration and discrimination of a predictive model for development of hypertensive disorders of pregnancy and/or fetal growth restriction within 1 week at <37 weeks' gestation using salivary uric acid measurements from the preceding 4 weeks and maternal demographics. AUC, area under the curve; CI, confidence intervals; CITL, Calibration‐In‐The‐Large; O:E, observed‐to‐expected ratio.

**FIGURE 2:**
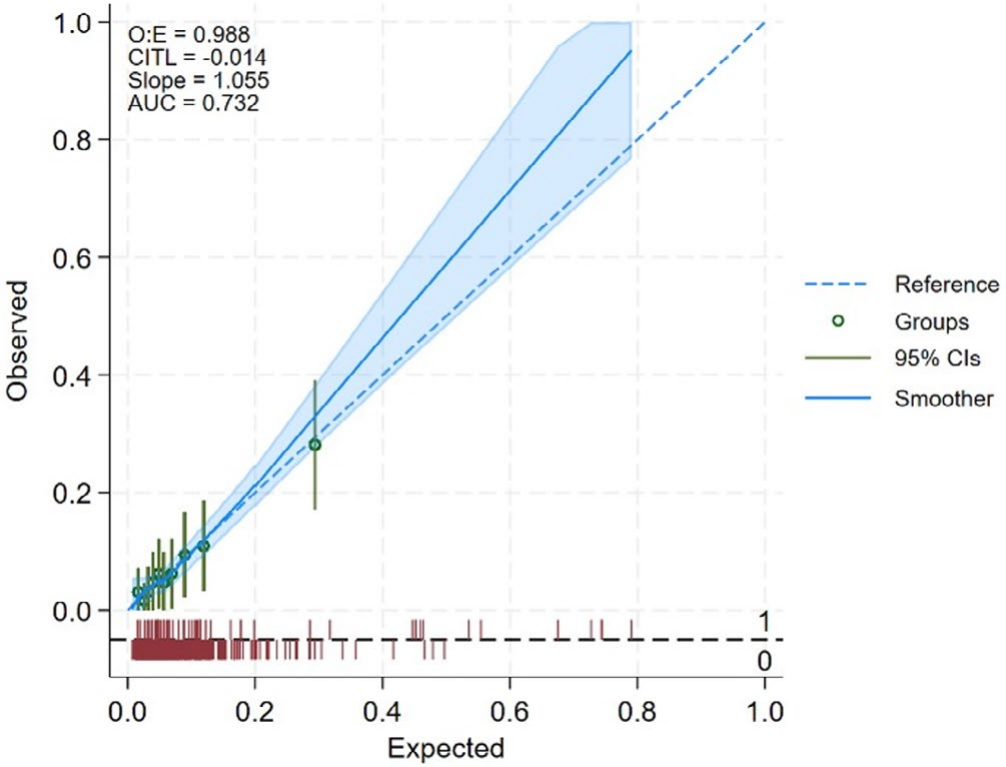
Prediction of term HDP/FGR within 1 week. Calibration and discrimination of a predictive model for development of hypertensive disorders of pregnancy and/or fetal growth restriction within 1 week at ≥37 weeks' gestation using salivary uric acid measurements from the preceding 4 weeks and maternal demographics. AUC, area under the curve; CI, confidence intervals; CITL, Calibration‐In‐The‐Large; O:E, observed‐to‐expected ratio.

The final multivariable logistic regression model included maternal BMI, maternal age, smoking status, prior PE, gestational week, and 4‐week lags of two colorimetric sUA values: RGB_mean and RGB_min (Table 2). Addition of serial RGB_mean and RGB_min values to maternal characteristics increased pseudo‐*R* ^2^ from 0.093 to 0.150 and AUC from 0.853 to 0.860. Models incorporating alternative RGB‐derived metrics demonstrated weaker performance (Figures S1 and S2). Using four preceding consecutive weeks' of sUA assessments we were able to generate individualized predictions for the development of signs or symptoms of uteroplacental insufficiency (HDP and/or FGR) within the following week. In‐sample evaluation demonstrated good predictive performance, with an AUC of 0.814 prior to 37 weeks (Figure 1) and an AUC of 0.732 after 37 weeks (Figure 2). The predictive performance of models using a 4‐week lag of tests leading up to 2‐ and 3‐weeks prior to an event showed similar performance (Figures S3–S6, Table S1).

**TABLE 2:** Modeling result for individual indices for the prediction of HDP and/or FGR within 1 week of the last test.

	Coefficient	Standard error	*z*	*p*‐value
RGB_mean				
L1	0.022 (0.003–0.042)	0.010	2.23	0.026
L2	−0.001 (−0.023 to 0.021)	0.011	−0.06	0.951
L3	−0.003 (−0.024 to 0.019)	0.011	−0.23	0.821
L4	−0.002 (−0.025 to 0.020)	0.012	−0.21	0.833
RGB_min				
L1	−0.029 (−0.050 to 0.001)	0.011	−2.77	0.006
L2	0.003 (−0.026 to 0.019)	0.011	−0.27	0.784
L3	−0.005 (0.028–0.017)	0.011	−0.47	0.642
L4	0.007 (−0.015 to 0.030)	0.012	0.63	0.525
Maternal age	−0.007 (−0.064 to 0.051)	0.029	−0.23	0.821
BMI	0.064 (0.022–0.107)	0.022	2.97	0.003
Smoking status	−1.41 (−4.25 to 1.42)	1.446	−0.98	0.328
Gestational age	0.269 (0.201–0.337)	0.035	7.71	0.000
Previous HDP	2.66 (1.99–3.33)	0.340	7.82	0.000
Constant	−14.4 (−18.1 to −10.7)	1.889	−7.63	0.000

Abbreviations: coefficient, estimated effect of each predictor on the log‐odds of the outcome and 95% confidence interval; FGR, fetal growth restriction; HDP, hypertensive disorder of pregnancy; L1‐L4, number of weeks lag of test prior to event; RGB_mean, mean RGB value of all pixels in individual tests; RGB_min, minimum RGB value of all pixels in individual tests; *z*, coefficient divided by its standard error.

## 4 DISCUSSION

In this prospective cohort study, we evaluated whether remote, serial, colorimetric self‐testing of sUA could contribute to the prediction of clinical manifestations of uteroplacental dysfunction. We found that color‐derived measurements of sUA demonstrated a measurable association with adverse pregnancy outcomes, and that a model incorporating weekly assessments of RGB metrics for 4 weeks, together with established maternal risk factors, provided moderate predictive performance.

There are currently no equivalent data supporting a serial, remote self‐test capable of monitoring evolving risk throughout pregnancy. Existing approaches are confined to first‐trimester prediction or to triage of symptomatic or already high‐risk women within clinical settings.^3^, ^4^, ^5^ A prediction model utilizing serial self‐measured sUA represents a novel approach, addressing the gap between early risk prediction and late clinical diagnosis. Püschl and colleagues first reported on sUA as a predictive test for HDP and FGR, demonstrating preliminary predictive potential in a pilot cohort, albeit in a smaller sample and without quantifying serial temporal patterns.^15^ More recent publications reinforce the concept that salivary markers can be feasibly measured remotely and independently by lay users, with an acceptable compliance rate.^16^ However, prior analyses largely focused on static or limited sampling rather than extended serial measurements. Our analysis builds on this by combining serial measurements and maternal characteristics in a mixed model analysis.

The model for adverse outcomes <37 weeks' gestation demonstrates good discrimination (AUC 0.814) but a tendency toward modest overprediction (observed‐to‐expected ratio 0.835). After 37 weeks, calibration improved substantially with Calibration‐In‐The‐Large (CITL) (−0.014) and observed‐to‐expected ratios (0.988) approaching ideal values, although discrimination declined (AUC 0.732). This pattern mirrors broader challenges in obstetric prediction modeling of identifying those at risk of preterm versus term HDP reflecting the different aetiological pathways and the effect of intervention bias on later‐onset HDP.^20^, ^21^, ^22^ A central feature of uteroplacental dysfunction is placental hypoxia and metabolic stress.^6^, ^7^ Uric acid has a well‐established relationship with these biological processes, exhibiting antioxidant properties at physiological concentrations and elevated levels during pro‐oxidant and pro‐inflammatory activity.^8^, ^9^ Serial changes in sUA may reflect evolving oxidative stress or microvascular dysfunction in the uteroplacental circulation before clinical manifestations. Colorimetric RGB measurements capture these changes, linking early placental dysfunction to changes in sUA prior to the diagnosis and development of clinical adverse outcomes.

Clinically, the concept of serial sUA acid remote monitoring could offer a noninvasive adjunct to current obstetric risk stratification, offering advanced notice and enabling timely access to healthcare and surveillance, especially in geographically underserved areas. In addition, it bridges the gap between complex multiparameter first‐trimester screening and the resources required for late pregnancy biochemical diagnostic triage. sUA self‐testing has the potential to provide noninvasive risk assessment throughout pregnancy using serial measurements that can be performed remotely. The models we propose can offer accurate risk assessment 1–3 weeks before adverse pregnancy outcomes, and further work is currently being undertaken to prospectively validate their predictive ability in a larger, multicenter and heterogenous cohort.^23^

The feasibility and acceptability of home‐based serial sampling are important strengths of this study. Over 300 women provided usable longitudinal data, demonstrating that weekly smartphone‐enabled monitoring can be accessed and sustained over many months. This could be particularly useful when access to laboratory testing or Doppler ultrasound is limited, or when individuals live far away from secondary care. However, despite the substantial sample size, the number of outcome events remained modest, particularly for early‐onset disease. Additionally, the cohort of participants was demographically homogenous, and further studies would need to focus on a more varied group of women. External validation for the predictive model is required.

## 5 CONCLUSION

Remote serial colorimetric measurement of maternal sUA offers a feasible, noninvasive approach to ongoing antenatal risk assessment for adverse pregnancy outcomes of uteroplacental dysfunction. In this cohort, RGB‐derived metrics of sUA combined with established maternal risk factors demonstrated moderate predictive performance for HDP and/or FGR. The ability to capture evolving biochemical changes remotely highlights its potential to identify at‐risk pregnancies earlier, supporting timely surveillance and intervention, especially in geographically underserved areas. Further studies are warranted to prospectively validate the model and explore whether predictive models could provide clinically useful lead times prior to clinical diagnosis.

## AUTHOR CONTRIBUTIONS

BC: data curation, methodology, writing original draft. CW: methodology, statistical analysis. NM: conceptualization, investigation, funding acquisition. AB: conceptualization, methodology, supervision. BT: conceptualization, methodology, review and editing, supervision.

## FUNDING INFORMATION

This work was supported by Innovate UK (Reference number 10053908), Region Zealand Health Sciences Research Foundation, and the Zealand University Hospital through the ReproHealth Research Consortium ZUH.

## CONFLICT OF INTEREST STATEMENT

No conflict of interest to declare by any of the authors.

## ETHICS STATEMENT

The study was approved by the Regional Committee on Health Research Ethics (SJ‐583 on March 6, 2017), the Danish Data Protection Agency (REG‐140‐2018), and the Danish Medicines Agency (2016100924). Study participants provided informed written consent.

## Supporting information


**Table S1.** Performance of prediction of HDP/FGR within 1, 2, and 3 weeks using salivary uric acid measurements from the preceding 4 weeks and maternal demographics.
**Figure S1**. Prediction of preterm HDP/FGR within 1 week using alternative RGB metrics. Calibration and discrimination of a predictive model for development of hypertensive disorders of pregnancy and/or fetal growth restriction within 1 week at <37 weeks' gestation, using salivary uric acid measurements from the preceding 4 weeks and maternal demographics with alternative RGB metrics: R_median, G_median and BL_median. AUC, area under the curve; CI, confidence intervals; CITL, Calibration‐In‐The‐Large; O:E, observed‐to‐expected ratio.
**Figure S2**. Prediction of term HDP/FGR within 1 week using alternative RGB metrics. Calibration and discrimination of a predictive model for development of hypertensive disorders of pregnancy and/or fetal growth restriction within 1 week at ≥37 weeks' gestation, using salivary uric acid measurements from the preceding 4 weeks and maternal demographics with alternative RGB metrics: R_median, G_median and BL_median. AUC, area under the curve; CI, confidence intervals; CITL, Calibration‐In‐The‐Large; O:E, observed‐to‐expected ratio.
**Figure S3**. Prediction of preterm FGR/HDP within 2 weeks. Calibration and discrimination of a predictive model for development of hypertensive disorders of pregnancy and/or fetal growth restriction within 2 weeks at <37 weeks' gestation using salivary uric acid measurements from the preceding 4 weeks and maternal demographics. AUC, area under the curve; CI, confidence intervals; CITL, Calibration‐In‐The‐Large; O:E, observed‐to‐expected ratio.
**Figure S4**. Prediction of term HDP/FGR within 2 weeks. Calibration and discrimination of a predictive model for development of hypertensive disorders of pregnancy and/or fetal growth restriction within 2 weeks at ≥37 weeks' gestation using salivary uric acid measurements from the preceding 4 weeks and maternal demographics. AUC, area under the curve; CI, confidence intervals; CITL, Calibration‐In‐The‐Large; O:E, observed‐to‐expected ratio.
**Figure S5**. Prediction of HDP/FGR within 3 weeks preterm. Calibration and discrimination of a predictive model for development of hypertensive disorders of pregnancy and/or fetal growth restriction within 3 weeks at <37 weeks' gestation using salivary uric acid measurements from the preceding 4 weeks and maternal demographics. AUC, area under the curve; CI, confidence intervals; CITL, Calibration‐In‐The‐Large; O:E, observed‐to‐expected ratio.
**Figure S6**. Prediction of HDP/FGR within 3 weeks at term. Calibration and discrimination of a predictive model for development of hypertensive disorders of pregnancy and/or fetal growth restriction within 3 weeks at ≥37 weeks' gestation using salivary uric acid measurements from the preceding 4 weeks and maternal demographics. AUC, area under the curve; CI, confidence intervals; CITL, Calibration‐In‐The‐Large; O:E, observed‐to‐expected ratio.

## Data Availability

The data that support the findings of this study are available from the corresponding author upon reasonable request.
