# Supplementary material for: Salivary uric acid remote serial self‐testing for prediction of adverse pregnancy outcomes of uteroplacental dysfunction
Source: Acta Obstet Gynecol Scand. 2026 May 14:10.1111/aogs.70257. Online ahead of print. doi: 10.1111/aogs.70257 (PMC13394806; doi:10.1111/aogs.70257)
Supplement: Supplementary file 1 — Table S1. Performance of prediction of HDP/FGR within 1, 2, and 3 weeks using salivary uric acid measurements from the preceding 4 weeks and maternal demographics. Figure S1. Prediction of preterm HDP/FGR within 1 week using alternative RGB metrics. Calibration and discrimination of a predictive model for development of hypertensive disorders of pregnancy and/or fetal growth restriction within 1 week at <37 weeks' gestation, using salivary uric acid measurements from the preceding 4 weeks and maternal demographics with alternative RGB metrics: R_median, G_median and BL_median. AUC, area under the curve; CI, confidence intervals; CITL, Calibration‐In‐The‐Large; O:E, observed‐to‐expected ratio. Figure S2. Prediction of term HDP/FGR within 1 week using alternative RGB metrics. Calibration and discrimination of a predictive model for development of hypertensive disorders of pregnancy and/or fetal growth restriction within 1 week at ≥37 weeks' gestation, using salivary uric acid measurements from the preceding 4 weeks and maternal demographics with alternative RGB metrics: R_median, G_median and BL_median. AUC, area under the curve; CI, confidence intervals; CITL, Calibration‐In‐The‐Large; O:E, observed‐to‐expected ratio. Figure S3. Prediction of preterm FGR/HDP within 2 weeks. Calibration and discrimination of a predictive model for development of hypertensive disorders of pregnancy and/or fetal growth restriction within 2 weeks at <37 weeks' gestation using salivary uric acid measurements from the preceding 4 weeks and maternal demographics. AUC, area under the curve; CI, confidence intervals; CITL, Calibration‐In‐The‐Large; O:E, observed‐to‐expected ratio. Figure S4. Prediction of term HDP/FGR within 2 weeks. Calibration and discrimination of a predictive model for development of hypertensive disorders of pregnancy and/or fetal growth restriction within 2 weeks at ≥37 weeks' gestation using salivary uric acid measurements from the preceding 4 weeks and maternal demogr [file AOGS-9999-0-s001.docx]

**Supplementary Material**

**TABLES**

**Table S1.** Performance of prediction of HDP/FGR within 1,2 and 3 weeks using salivary uric acid measurements from the preceding 4 weeks and maternal demographics..

|  | O:E | CITL | AUC |
| --- | --- | --- | --- |
| Prediction of preterm HDP/FGR within 1 week | **0.835** | **-0.200** | **0.814** |
| Prediction of preterm HDP/FGR within 2 weeks | **0.841** | **-0.190** | **0.798** |
| Prediction of preterm HDP/FGR within 3 weeks | **0.865** | **-0.158** | **0.795** |
| Prediction of term HDP/FGR within 1 week | **0.988** | **-0.014** | **0.732** |
| Prediction of term HDP/FGR within 2 weeks | **0.995** | **-0.006** | **0.724** |
| Prediction of term HDP/FGR within 3 weeks | **0.987** | **-0.016** | **0.728** |

O:E = observed-to-expected ratio. CITL = Calibration-In-The-Large. AUC = area under the curve.

**FIGURES**

**
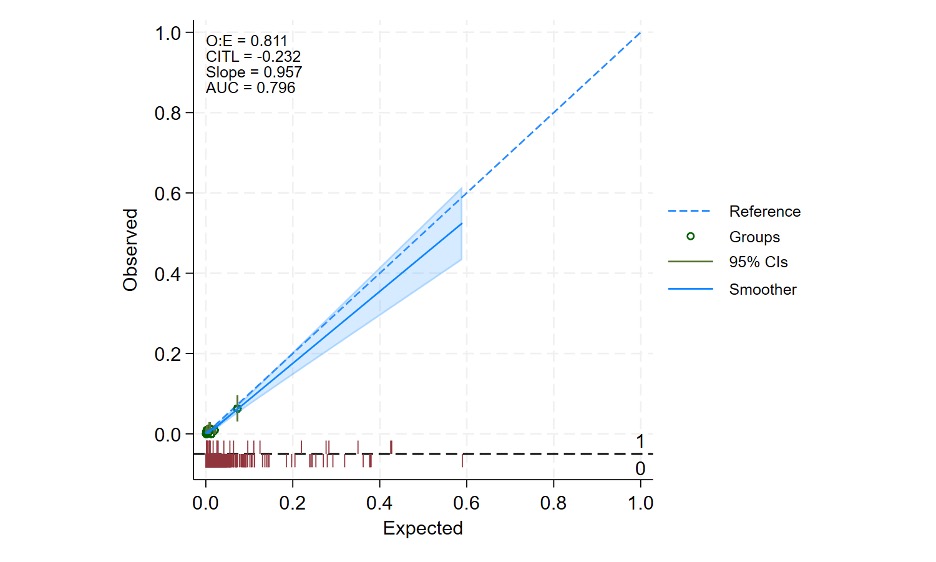
**

**Figure S1.** Prediction of preterm HDP/FGR within 1 week using alternative RGB metrics. Calibration and discrimination of a predictive model for development of hypertensive disorders of pregnancy and/or fetal growth restriction within 1 week at <37 weeks’ gestation, using salivary uric acid measurements from the preceding 4 weeks and maternal demographics with alternative RGB metrics: R_median, G_median and BL_median.

O:E = observed-to-expected ratio. CITL = Calibration-In-The-Large. AUC = area under the curve. CI = confidence intervals.

**
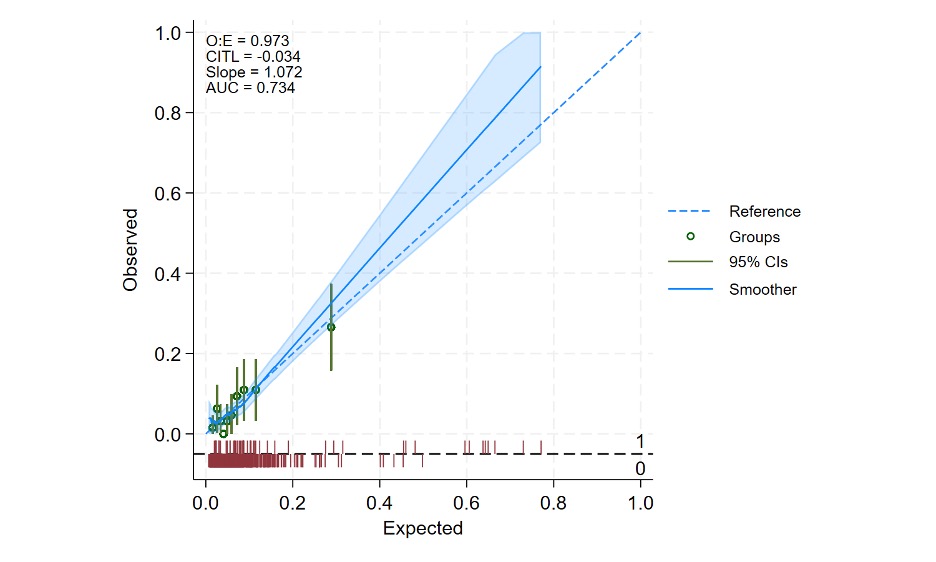
**

**Figure S2.** Prediction of term HDP/FGR within 1 week using alternative RGB metrics. Calibration and discrimination of a predictive model for development of hypertensive disorders of pregnancy and/or fetal growth restriction within 1 week at ≥37 weeks’ gestation, using salivary uric acid measurements from the preceding 4 weeks and maternal demographics with alternative RGB metrics: R_median, G_median and BL_median.

O:E = observed-to-expected ratio. CITL = Calibration-In-The-Large. AUC = area under the curve. CI = confidence intervals.


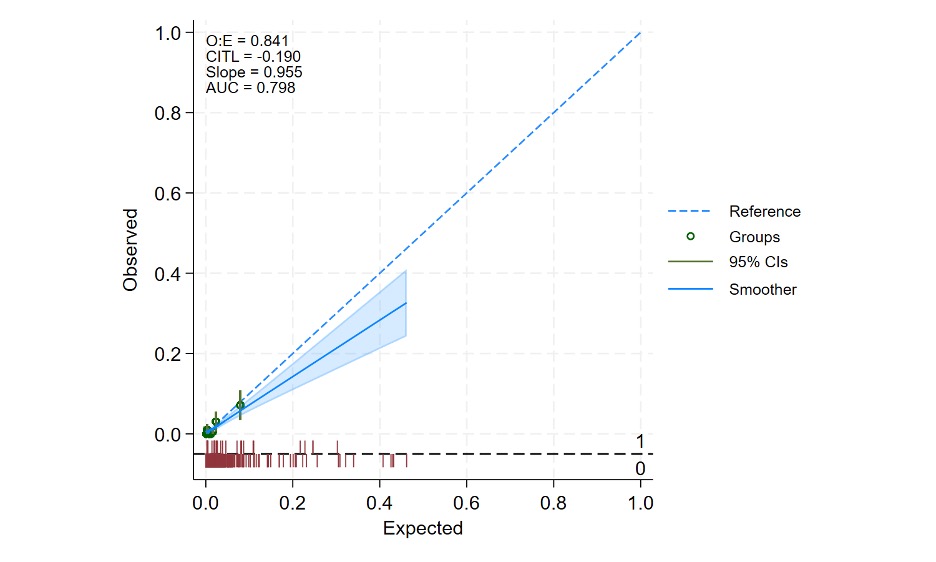


**Figure S3.** Prediction of preterm FGR/HDP within 2 weeks. Calibration and discrimination of a predictive model for development of hypertensive disorders of pregnancy and/or fetal growth restriction within 2 weeks at <37 weeks’ gestation using salivary uric acid measurements from the preceding 4 weeks and maternal demographics.

O:E = observed-to-expected ratio. CITL = Calibration-In-The-Large. AUC = area under the curve. CI = confidence intervals.

**
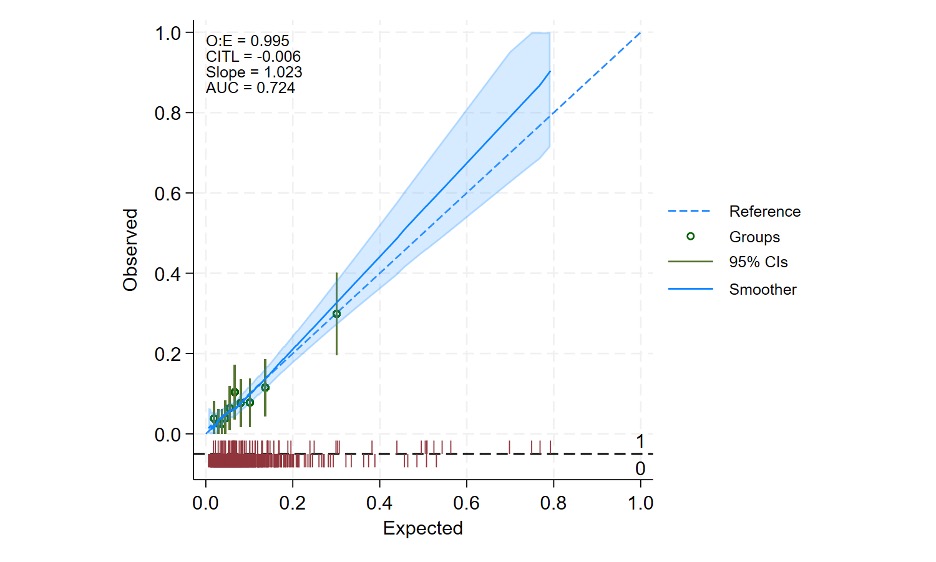
**

**Figure S4.** Prediction of term HDP/FGR within 2 weeks. Calibration and discrimination of a predictive model for development of hypertensive disorders of pregnancy and/or fetal growth restriction within 2 weeks at ≥37 weeks’ gestation using salivary uric acid measurements from the preceding 4 weeks and maternal demographics.

O:E = observed-to-expected ratio. CITL = Calibration-In-The-Large. AUC = area under the curve. CI = confidence intervals.

**
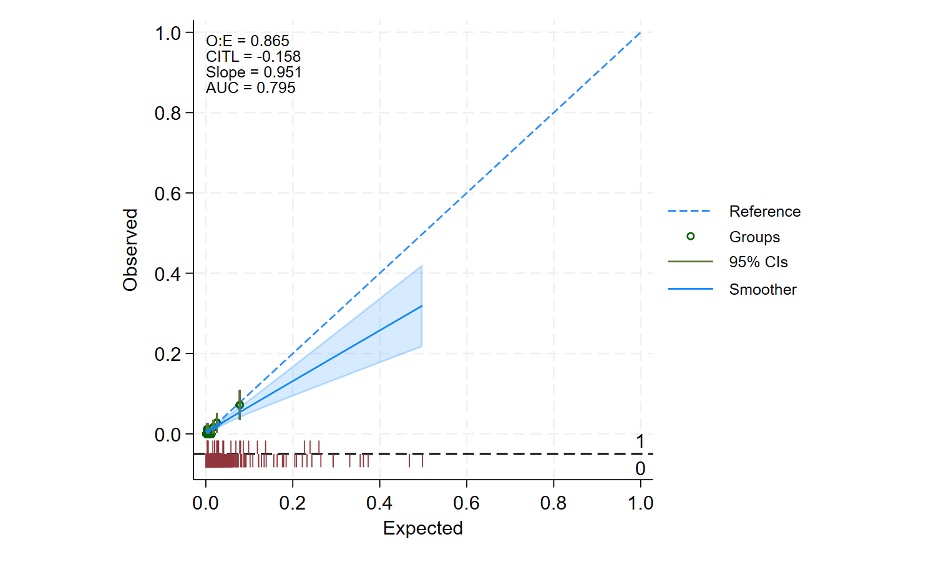
**

**Figure S5.** Prediction of HDP/FGR within 3 weeks preterm. Calibration and discrimination of a predictive model for development of hypertensive disorders of pregnancy and/or fetal growth restriction within 3 weeks at <37 weeks’ gestation using salivary uric acid measurements from the preceding 4 weeks and maternal demographics.

O:E = observed-to-expected ratio. CITL = Calibration-In-The-Large. AUC = area under the curve. CI = confidence intervals.

**
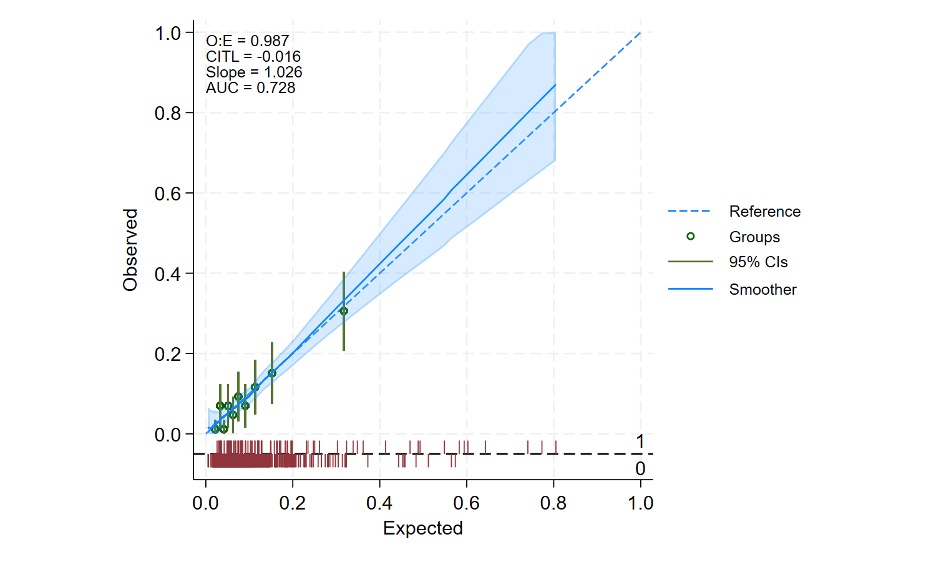
**

**Figure S6.** Prediction of HDP/FGR within 3 weeks at term. Calibration and discrimination of a predictive model for development of hypertensive disorders of pregnancy and/or fetal growth restriction within 3 weeks at ≥37 weeks’ gestation using salivary uric acid measurements from the preceding 4 weeks and maternal demographics.

O:E = observed-to-expected ratio. CITL = Calibration-In-The-Large. AUC = area under the curve. CI = confidence intervals.
